# Transdifferentiation of Integrin Beta 1 High+ Skin Progenitor Cells Into Functional Hepatocytes

**DOI:** 10.1155/sci/8953305

**Published:** 2025-04-23

**Authors:** Jung Hwa Lim, Dae Hun Kim, Junhee Lee, Cho-Rok Jung, Hyun Mi Kang

**Affiliations:** ^1^Stem Cell Convergence Research Center, Korea Research Institute of Bioscience and Biotechnology (KRIBB), Daejeon 34141, Republic of Korea; ^2^Korea University of Science and Technology (UST), Daejeon, Republic of Korea; ^3^Department of Bionic Machinery, Research Institute of AI Robotics, Korea Institute of Machinery and Materials (KIMM), Daejeon 34103, Republic of Korea

**Keywords:** albumin secretion, drug metabolism, epidermal progenitors, hepatocytes

## Abstract

A highly reproducible and functional liver model that closely resembles the human liver plays a crucial role in drug development, disease research, personalized medicine, and regenerative medicine. This study aimed to establish an in vitro liver model using skin epidermal progenitor cells (EPCs), which are easily accessible and exhibit a high proliferative capacity. Skin EPCs with high integrin beta 1 expression demonstrated multipotent differentiation potential, capable of differentiating into adipocyte- and neuron-like cells in vitro. Furthermore, when exposed to high concentrations of activin A, along with Wnt3a and BMP4, these cells efficiently differentiated into definitive endoderm, exhibiting high FOXA2 expression. Under our culture conditions, they further differentiated into functional hepatocytes. These differentiated cells exhibited high albumin secretion, CYP activity, and drug metabolism capabilities similar to those observed in vivo. In conclusion, this study highlights the potential of EPCs to differentiate into functional hepatocytes, providing a feasible and scalable source of hepatocytes for drug screening, liver disease modeling, and potential cell-based therapies.

## 1. Introduction

The liver is a vital organ responsible for a myriad of physiological functions, including metabolism, detoxification, and the synthesis of essential proteins. Hepatocytes, the primary parenchymal cells of the liver, play a central role in maintaining these functions. Dysfunction or loss of hepatocytes can lead to severe liver diseases, such as cirrhosis and hepatocellular carcinoma, highlighting the critical need for effective therapeutic strategies [1]. Since hepatotoxicity is the main cause of drug withdrawal after postmarketing, in vitro liver models are urgently needed to test the safety and efficacy of new drugs during the drug development process [2]. In addition, these in vitro liver models play a crucial role in advancing our understanding of liver biology, drug metabolism, and disease mechanisms, ultimately contributing to the development of safer and more effective therapies for patients.

In this context, scientists have shown great interest in in vitro hepatocyte modeling. Primary human hepatocytes (PHHs) are widely regarded as the gold standard for hepatic models due to their ability to accurately represent human liver metabolism and functionality [3]. Due to the challenges associated with obtaining and expanding PHH, significant efforts have been made over the past decades to develop in vitro liver models derived from various sources, including pluripotent stem cells (PSCs), for the study of liver biology, drug metabolism, and disease mechanisms, ultimately leading to safer and more effective therapies for patients [4–7]. Despite their high potential, PSC-derived hepatocytes still have limitations such as immaturity in cell phenotype and functionality, leading to the development of alternatives to these cells.

Several investigations have reported on the epidermal stem cell populations in the primary skin cells using integrins, G protein-coupled receptors, cytokeratins, transcription factors, cadherins, and catenins [8–11], as well as their multipotent differentiation properties in vitro [12, 13]. In a previous study, we established stable epidermal progenitor cells (EPCs) expressing high levels of ITGB1, showing high proliferative capacity and the potential to develop a functional skin model in vitro and in vivo [14]. Moreover, these cells have the potential to differentiate into mesodermal lineage cells such as adipocytes, osteoblasts, and chondrocytes, as well as skin epidermis.

Therefore, in this study, we attempted to elucidate the possibility of transdifferentiation of skin EPCs into hepatocytes. Furthermore, in a previous study, we observed that the expression of FOXA2 could facilitate hepatic differentiation in hepatoblast cells [15]. Based on this finding, we sought to convert EPCs into definitive endoderm expressing FOXA2, and under our culture conditions, these cells successfully differentiated into functional hepatocytes.

## 2. Materials and Methods

### 2.1. Antibodies and Reagents

CD29 (abcam, #ab24693, Cambridge, UK), Albumin (Santa Cruz Biotechnology, #sc271604, Texas, USA), p63 (abcam, #ab111683), YAP1 (Cell Signaling Technology, #14074S, Massachusetts, USA), CYP3A4 (Cell Signaling Technology, #13384S), SOX17 (Cell Signaling Technology, #13963), FOXA2 (abcam, #ab108422), tubulin III (TUBB3) (abcam, #ab18207), neuronal nuclear (NeuN) protein NeuN (Cell Signaling Technology, #24307S) dexamethasone (Sigma, St. Louis, Missouri, USA), human insulin (Sigma, St. Louis, Missouri, USA), insulin-transferrin-selenium (ITS, Gibco), bovine serum albumin (BSA, Sigma, St. Louis, Missouri, USA), KGM-gold (Lonza, Basel, Switzerland), RPMI-1640 (Hyclone, South Logan, Utah, USA), and William's E medium (Gibco).

### 2.2. EPCs Culture

Human EPCs were cultured through previously described methods [14]. Human primary keratinocyte purchased from Biosolution (#HEK-A/F, Seoul, Korea) and ATCC cultured in KGM-gold medium onto a 0.2% gelatin-coated culture dish at 5% CO2, at 37°C. For isolation of EPCs, cells were sorted using FITC-conjugated anti-CD29 antibody. After 5–7 days, cells (at 80% confluence) were detached using 0.5% Trypsin-EDTA and passaged.

### 2.3. Quantitative Real-Time Polymerase Chain Reaction (qPCR)

RNA was isolated using the RNAeasy Mini kit, 1 μg was reverse transcribed using the cDNA archival kit (Life Technologies, Gaithersburg, MD), and qPCR was performed according to the manufacturer's instructions (Agilent Technologies, Santa Clara, CA) using SYBRGreen Master Mix. The data were normalized and analyzed using the *ΔΔ*CT method. Primers used are listed in Table S1.

### 2.4. Colony-Forming Unit Assay

Primary keratinocytes or EPCs (1 × 10^3^ cells) were plated onto six-well culture dishes. On day 10, cells were fixed with methanol and stained using crystal violet staining solution (5 min at room temperature). After washing and mounting, colonies between 1 and 8 mm in diameter (more than 20 cells) were counted.

### 2.5. Assessment of Multilineage Differentiation Potential

To induce adipogenic differentiation, cells (at passage 3) were cultured in an adipogenic medium consisting of RPMI-1640, 10% FBS, 1 μM dexamethasone, 0.5 μM 3-isobutyl-1-methylxanthine, 0.05 mg/mL human insulin, and 60 μM indomethacin. After 14 days of culture, cells were stained using Oil Red O for the presence of intracellular lipid droplets, indicative of adipocytes. For neuronal differentiation, cells were seeded into a culture dish at a concentration of 3500/cm^2^ with basal medium (Advanced DMEM/F12 supplemented with 1x glutamax). After 24 h, the medium was replaced by neural differentiation medium based on basal medium supplemented with 1 mM dbcAMP, 0.5 mM IBMX, 20 ng/mL hEGF, 40 ng/ml bFGF, 10 ng/mL FGF8, and 10 ng/mL BDNF, and cells were cultured for additional 10 days.

### 2.6. Definitive Endoderm Inducting and Hepatocyte Differentiation

To induce the definitive endoderm expressing FOXA2, cells (at passage 3–10) were cultured in definitive endoderm medium consisting of RPMI-1640 supplemented with 1x B27 supplement, 100 ng/mL activin A, 3 *μ*M CHIR99021, 25 ng/mL Wnt3a, and/or 10 ng/mL BMP4 for 7 days. After 7 days, cells were cultured in the hepatocyte induction medium consisting of RPMI-1640 supplemented with 1x B27 supplement, 1x ITS, 5 μM A8301, 1 mM nicotinamide, 100 nM dexamethasone, 20 ng/mL OSM, 25 ng/mL HGF, and 10 ng/mL bFGF for 14 days.

### 2.7. Immunofluorescence and Immunocytochemical Analysis

Cells were fixed with 4% paraformaldehyde and incubated in 2% goat or horse serum with 0.2% fish skin gelatin at room temperature for 1 h to block nonspecific binding. Then cells were incubated with primary antibodies overnight at 4 °C, and then secondary antibodies at room temperature for 1 h. Nuclei were counterstained with 4',6-diamidino-2-phenylindole.

### 2.8. Flow Cytometry

Primary keratinocyte EPSCs were harvested by trypsinization, washed, and resuspended in PBS supplemental with 2% FBS. Fluorescein conjugate primary antibody incubation was applied for 30 min at 4 °C and rinsed, and resuspended in PBS. All data acquisition was performed on a FACS Aria or FACS Calibur flow cytometer (BD Biosciences) with Cell Quest Pro software.

### 2.9. Operation of a Networking Cell Culture System (NCCS) for Drug Metabolism and Analysis of Samples

The NCCS was used to validate the functionality of differentiated hepatocytes through drug metabolism, as described in our previous study [15]. On the NCCS, intestinal cells (Caco-2) were cultured in a Transwell-type dish, while differentiated hepatocytes were prepared in a mashed-side well-type dish. Caco-2 cells (1 × 10^6^ cells/well) were seeded into a transwell-type dish and cultured in RPMI-1640 medium containing 5% FBS and penicillin/streptomycin for 14. In addition, differentiated HepaRG cells were used for a positive control. HepaRG (2 × 10^6^ cells/well) cells were seeded into a mashed-side type dish and then cultured with William's E medium containing 5% FBS and penicillin/streptomycin for 7 days, and 2% of DMSO was added to the medium for an additional 23 days as manufacturer's guidance. These two types of dishes were integrated, and the culture medium was circulated using a fluidic controller. The NCCS was set at a flow rate of 2 mL/min and a shaking speed of 0.8 revolutions per second for 10 min. The test drug was applied to the apical side of the intestinal cells at an appropriate concentration and subsequently transported to the hepatocytes. Medium samples were collected from the connected tubing after the liver unit (the last unit of the series) at each time point. Media samples were analyzed using liquid chromatography with tandem mass spectrometry.

### 2.10. Statistical Analyses

The data are presented as means ± SEM. Unpaired Student's *t*-test was used for comparisons between two groups. We used one-way ANOVA with Tukey's post hoc tests to compare multiple groups. A *p*-value < 0.05 or < 0.01 was considered to be significant.

## 3. Results

### 3.1. EPCs Showed Stem Cell-Like Properties In Vitro

**Figure 1:**
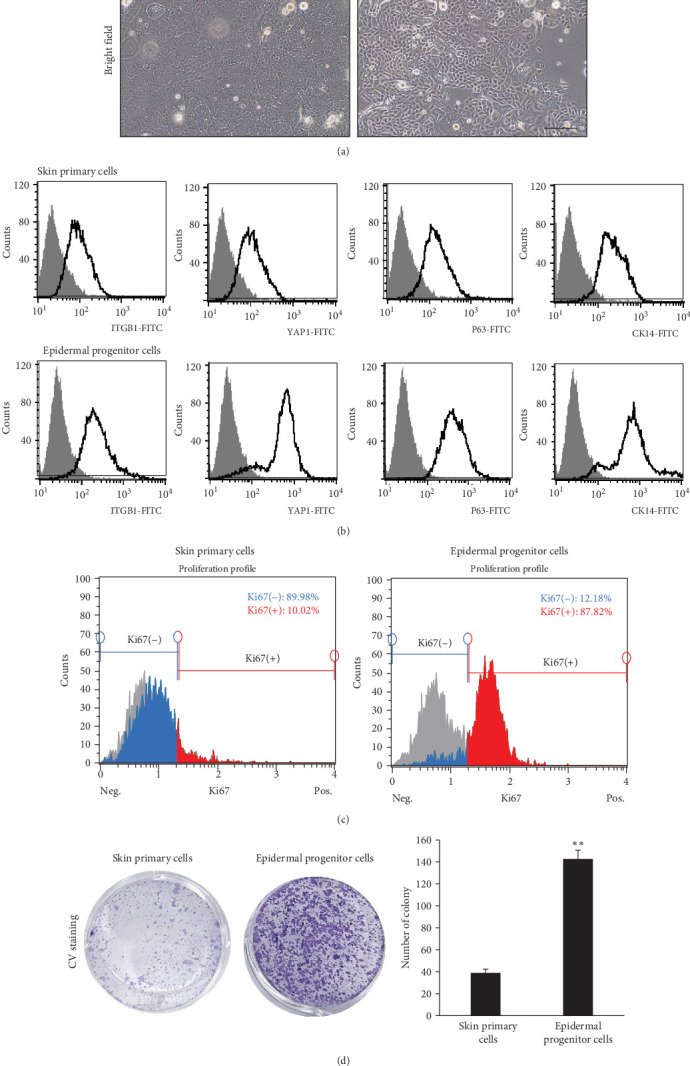
Stem cells-related properties of epidermal progenitors derived from human skin primary cells, (A) morphology of human skin primary cells at early passage (P2) and epidermal progenitor cells (EPCs) at passage 10 in vitro, (B) FACS analysis of skin stem cell markers of skin primary cells and EPCs at passage 3, (C) FACS analysis of cell proliferative potential of skin primary cells and EPCs at passage 4, and (D) Representative images (left) and number of colony (right) of crystal violet stained colonies of skin primary cells and EPCs at passage 4. All data are depicted as mean ± SEM. *⁣*^*∗∗*^*p* < 0.01 compared to skin primary cells via unpaired Student's *t*-test. Scale bar, 100 μm.

**Figure 2:**
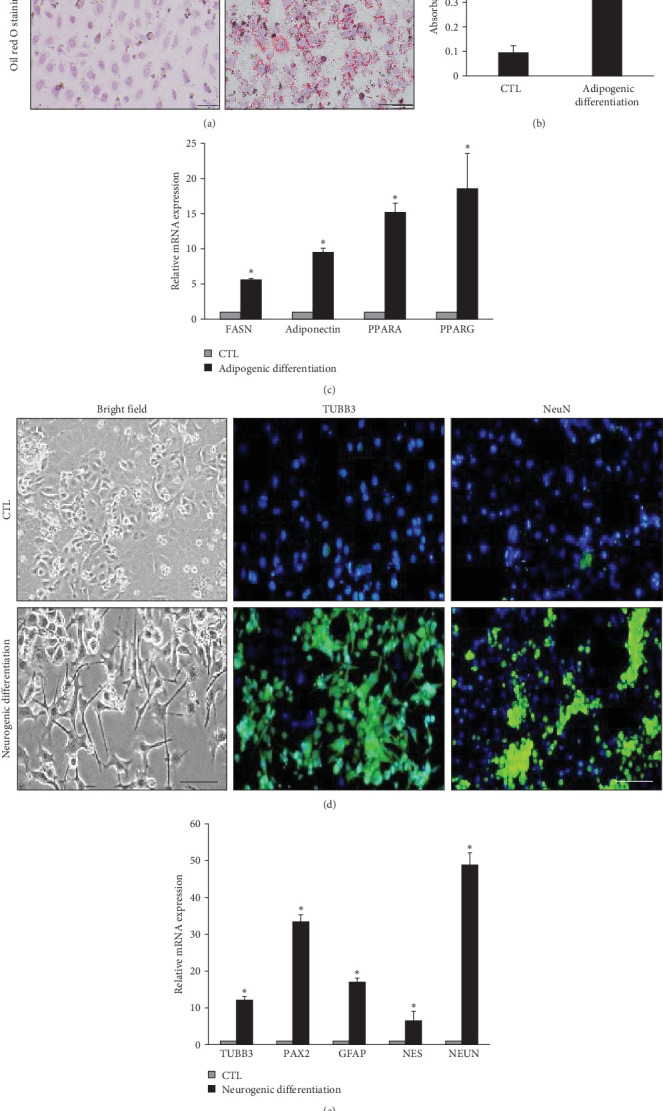
Meso- and ectodermal lineage differentiation potential of epidermal progenitor cells (EPCs) in vitro. (A) and (B) representative image of adipogenic differentiation of EPCs assessed via Oil red O staining (A) and quantification of Oil red O (B), (C) transcript levels of adipocyte markers, (D) representative image of neurogenic differentiation of EPSCs assessed via cell morphology analysis, beta tubulin III (TUBB3), and neuronal nuclear (NeuN) protein immunostaining, and (E) transcript levels of neuronal markers. All data are depicted as mean ± SEM *⁣*^*∗*^*p* < 0.05 compared to CTL via unpaired Student's *t*-test. Scale bar, 100 μm.

In our previous study, we isolated EPCs that showed highly proliferative and multipotent differentiation potential [14]. To induce these EPCs into functional hepatocytes, we first analyzed whether these cells possess stem cell-related characteristics. When skin primary cells changed their morphology and lost the proliferative capacity at passage 5, but EPCs maintained their initial epithelial cell morphology and proliferative potential at passage 10 (Figure 1A). These cells highly expressed epidermal stem cell markers, such as ITGB1, YAP1, P63, and CK14, compared to primary skin cells (Figure 1B) as well as the proliferation marker Ki67 (Figure 1C). They also exhibited clonal expansion potential for 2 weeks of culture with extremely low cell density compared to the skin primary cells at passage 4 (Figure 1D). To assess the multipotent differentiation potential, cells cultured in an adipogenic medium for 2 weeks exhibited intense cytoplasmic staining for Oil Red O at passage 5 (Figure 2A), indicating lipid accumulation, along with the upregulation of adipocyte-related genes and adipocyte-related gene expression (Figure 2B). They also exhibited ectodermal differentiation into neuron-like cells, as evidenced by their neuronal morphology and neuronal marker expression, such as that of TUBB3 and NeuN, when cultured for 2 weeks in a neurogenic medium (Figure 2C,D). These results suggest that EPCs possessed high proliferative capacity and multipotent differentiation potential in vitro. Based on these findings, we attempted to differentiate these cells into functional hepatocytes.

### 3.2. Transdifferentiation of Epidermal Progenitors Into Definitive Endoderm

**Figure 3:**
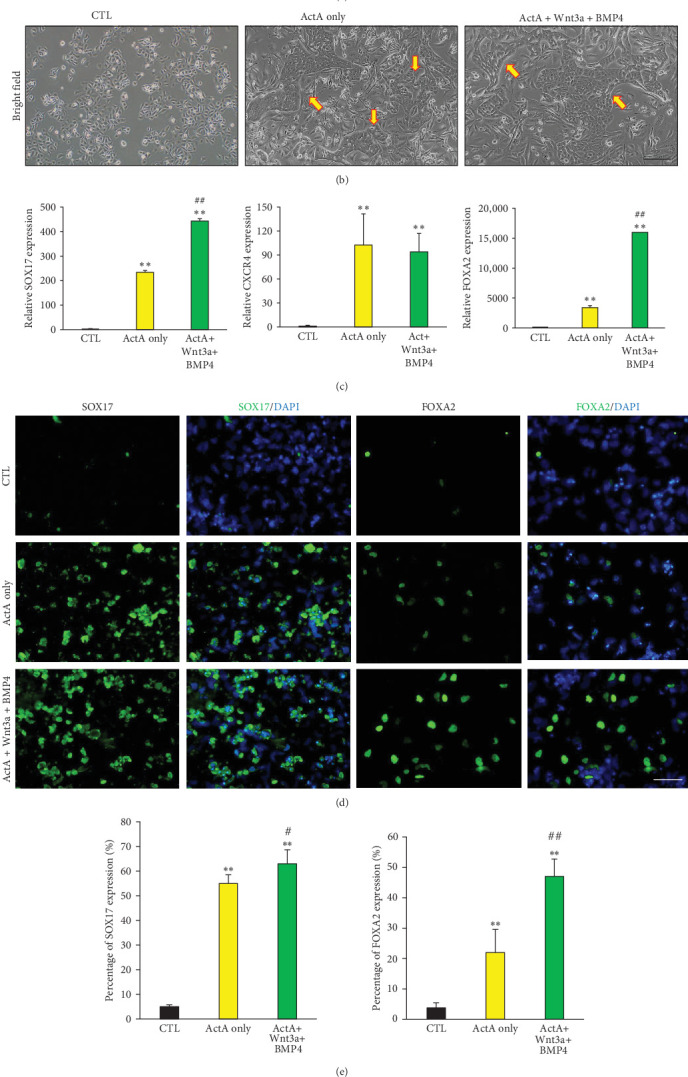
Definitive/hepatic endoderm differentiation of epidermal progenitor cells (EPCS). (A) a schematic showing the definitive endoderm and hepatocyte differentiation protocol, (B) morphology of definitive endoderm cells cultured with activin A and/or Wnt3a and BMP4 for 7 days. Arrows indicated the differentiated cells tended to change in cobblestone like morphology, (C) transcript levels of definitive/hepatic endoderm related markers, (D) representative image of definitive/hepatic endoderm differentiation of epidermal progenitors via SOX17 and FOXA2 expression, and (E) quantification of SOX17 and FOXA2 stained cells. All data are depicted as mean ± SEM. *⁣*^*∗∗*^*p* < 0.01 compared to CTL and  ^#^^#^*p* < 0.01 compared to Act A only treated cells via unpaired Student's *t*-test. Scale bar, 100 μm.

To induce the differentiation of ectoderm-derived EPCs into endodermal hepatocytes, we mimicked the developmental processes of hepatic differentiation in vivo (Figure 3A). First, EPCs were treated with various factors to induce their differentiation into definitive endoderm cells. Upon exposure to a high concentration of activin A, the morphology of the differentiated cells tended to change to a cobblestone-like morphology (Figure 3B, arrow). Quantitative reverse transcription-PCR (qRT-PCR) results showed an increase in the expression of definitive endoderm markers such as Sox17, CXCR4, and FOXA2 (Figure 3C). However, immunostaining showed a significant increase in Sox17 expression, while Foxa2 expression was observed in only approximately 20% of the cells (Figure 3D,E). FOXA2, also known as hepatocyte nuclear factor 3 beta, is a transcription factor that plays a crucial role in liver development, and previous research indicated that increased FOXA2 expression is involved in the direct conversion of fibroblasts into hepatocytes [15]. Additionally, it has been reported to play a crucial role in the hepatic differentiation of hepatic progenitor cells. Therefore, to establish a differentiation condition that effectively induces FOXA2 expression from definitive endoderm cells for efficient hepatocyte differentiation, we added high concentrations of activin A along with WNT3A and BMP4, known to be effective in definitive endoderm differentiation. FOXA2 expression was significantly upregulated in the cells cultured with all the factors compared to those with activin A only (Figure 3C). To further corroborate these findings, we performed immunohistochemistry to assess SOX17 and FOXA2 expression in definitive endoderm cells. Strong staining for both proteins was observed, with the most robust staining detected in cells treated with activin A, WNT3A, and BMP4 (Figure 3D,E). These data suggest that under the culture conditions described in this study, EPCs could effectively transdifferentiate into definitive endoderm.

### 3.3. Generation of Functional Hepatocytes via the Definitive Endodermal Stage Derived From EPCs

**Figure 4:**
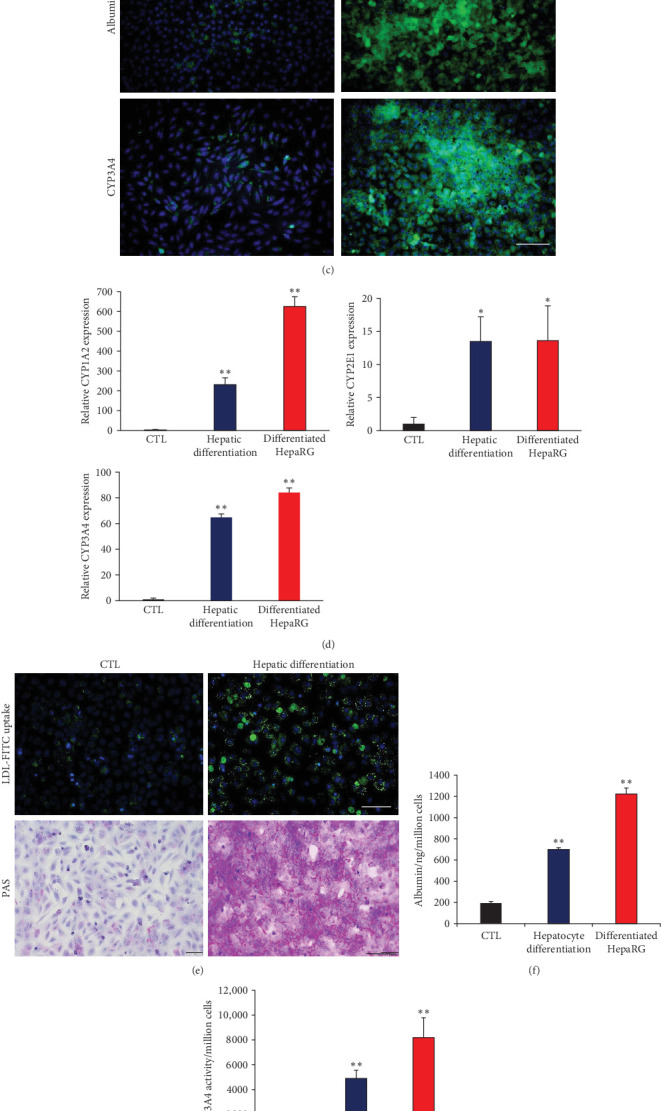
Hepatic differentiation of epidermal progenitor cells (EPCs). (A) representative images of hepatic differentiation of EPCs, (B) transcript levels of hepatocyte markers in differentiated cells derived EPCs. Differentiated hepaRG was used as a positive control, (C) representative stained images with albumin and CYP3A4 of hepatic differentiation derived from EPCs, (D) relative mRNA expression related to drug metabolism of hepatocytes in differentiated cells. Differentiated hepaRG was used as a positive control, (E–G) LDL uptake and glycogen storage (E), albumin secretion (F), and CYP3A4 enzyme activity (G) of differentiated cells. Differentiated hepaRG was used as a positive control, and (H) FACS analysis for hepatic markers expression in differentiated cells derived EPCs. All data are depicted as mean ± SEM. *⁣*^*∗*^*p* < 0.05 and *⁣*^*∗∗*^*p* < 0.01 compared to CTL via unpaired Student's *t*-test. Scale bar, 100 μm.

**Figure 5:**
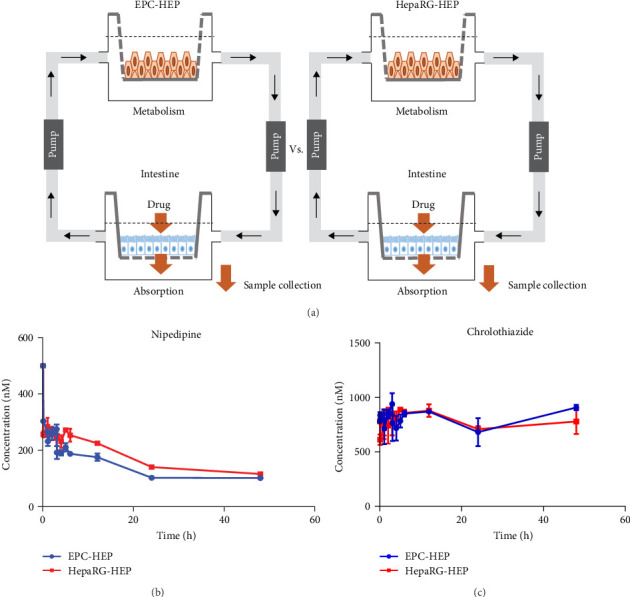
Drug metabolism profiles of hepatic differentiated cells. (A) Illustration of networking cell culture system (NCCS). Intestinal cells (Caco-2) and differentiated hepatocytes derived from epidermal progenitor cells (EPC-HEP) or hepaRG (hepaRG-HEP) are located on the multi-insert culture dish for drug evaluation, (B) and (C) metabolism profile of nipedipine (B) and chrolothiazide (C) of hepatic differentiated cells derived from epidermal progenitor cells (EPC-HEP) and hepaRG (HepaRG-HEP) for 48 h. The graph shows the changes of concentration of the drug for 48 h. Differentiated hepaRG (HepaRG-HEP) was used for a positive control.

Differentiated cells changed from epithelial cells to hepatocyte-like cells at 10 days of differentiation (Figure 4A). qRT-PCR results showed that the expression of hepatocyte-related markers such as ALB, HNF4a, and ALDOB, as well as FOXA2, were significantly increased in our hepatocyte-like cells and expression levels were similar to the differentiated hepaRG cells, a hepatic cell line that retains many characteristics of primary hepatocytes (Figure 4B). Immunostaining further confirmed the increased expression of the hepatic marker albumin (Figure 4C). In addition, transcripts of three cytochrome P450 proteins (CYP1A2, 2E1, and 3A4) were analyzed by qRT-PCR in the differentiated cells, and CYP3A4 expression was significantly increased, similar to the differentiated hepaRG (Figure 4D), and protein expression was also confirmed in the differentiated cell (Figure 4C). To validate the functionality of the differentiated hepatocytes, we assessed LDL uptake, glycogen storage, albumin secretion, and CYP3A4 activity. The differentiated hepatocytes were capable of taking up LDL present in the medium, and glycogen storage was confirmed through PAS staining (Figure 4E). In addition, albumin secretion and CYP3A4 activity were also significantly increased in the differentiated cells (Figure 4F,G). Flow cytometry analysis revealed a significant increase in the expression of hepatocyte-specific markers, including albumin, AAT, and HNF4A, compared to undifferentiated cells (Figure 4H). In our previous study, we found that drug metabolism patterns in cells were more similar to in vivo drug metabolism profiles when drugs were administered under circulating culture conditions rather than direct treatment under static conditions [15]. In our previous study, we observed that, compared to static drug treatment, dynamic circulation of the culture medium led to drug absorption and metabolism patterns that more closely resembled in vivo conditions [15]. Based on these findings, we utilized the NCCS to validate the functionality of differentiated hepatocytes in drug metabolism. To assess whether the differentiated cells could metabolize drugs, we prepared intestinal cells (Caco-2) and differentiated hepatocytes and treated them with nifedipine and chlorothiazide, drugs with different metabolic rates in vivo. The drugs were applied to the apical side of the intestinal cells, and absorbed drugs were subsequently transported to the hepatocytes (Figure 5A). Drug concentrations in the hepatocyte culture medium were monitored over 48 h. The results showed that the concentration of nifedipine began to decrease 30 min after treatment, whereas chlorothiazide showed little change in concentration over the 48-h period (Figure 5B,C). Furthermore, these drug metabolism responses were similar to those observed in differentiated HepaRG cells, a well-established in vitro liver model (Figure 5B,C). These findings suggest that the differentiated hepatocytes exhibit high functionality in drug metabolism, similar to in vivo liver cells. Collectively, these observations indicated that epithelial progenitor cells could differentiate into functional hepatocytes under our culture conditions.

## 4. Discussion

The successful induction of hepatocyte differentiation from skin EPCs represents a significant advancement in regenerative medicine and tissue engineering. Our study highlights the plasticity of EPCs and their ability to differentiate into endodermal lineages, particularly hepatocytes, under defined culture conditions. A key finding of our study is the ability of specific culture conditions to effectively induce the differentiation of EPCs into definitive endoderm, as demonstrated by the expression of hepatic markers like FOXA2. This suggests that epidermal progenitors possess the inherent capacity to undergo lineage conversion toward hepatic fate when exposed to appropriate signaling cues. FOXA2, a key transcription factor, plays a crucial role in hepatocyte differentiation, as evidenced by its significant upregulation during liver development and its essential functions in hepatic gene regulation [16–18]. In our study, the induction of FOXA2 expression proved to be pivotal in promoting the differentiation of progenitor cells into functional hepatocytes. This underscores the importance of FOXA2 in orchestrating the molecular events necessary for hepatocyte specification and maturation. FOXA2 induction seems to trigger a cascade of molecular events driving progenitor cell differentiation toward a hepatic lineage [19]. FOXA2 functions as a pioneer factor, facilitating chromatin remodeling and enabling the binding of other transcription factors essential for hepatocyte identity [20]. Its expression not only marks the commitment of progenitor cells to a hepatic fate but also regulates the expression of key hepatic genes involved in metabolic functions, detoxification pathways, and bile acid synthesis [21–24]. In this study, we attempted to transdifferentiate ectoderm-derived epidermal progenitors into hepatocytes via definitive endodermal cells expressing FOXA2. These FOXA2-expressing definitive endoderm cells successfully differentiated into functional hepatocytes, secreting albumin and exhibiting CYP3A4 activity. Previous studies have demonstrated that FOXA2 induction in bipotent hepatic progenitor cells promotes hepatocyte differentiation [15]. Moreover, FOXA2 serves as a critical determinant in the fate specification of bipotent hepatocyte progenitor cells. Its expression and activity regulate the balance between hepatocyte and cholangiocyte differentiation pathways. FOXA2 not only activates hepatocyte-specific genes but also represses cholangiocyte-specific gene expression [25], thereby directing progenitor cells toward a hepatocyte fate. Furthermore, the ability to derive hepatocytes from EPCs offers several advantages over traditional cell sources. EPCs are abundant and easily accessible, reducing the ethical concerns and logistical challenges associated with other cell sources such as embryonic stem cells or induced PSCs. Moreover, their noninvasive isolation and ease of expansion make them promising candidates for personalized cell-based therapies. However, several questions and challenges remain. Further investigation is needed to elucidate the molecular mechanisms underlying the conversion of EPCs into hepatocytes and to optimize differentiation protocols for greater efficiency and reproducibility. Additionally, long-term studies are necessary to assess the stability and functionality of the differentiated hepatocytes in vitro and in vivo.

In conclusion, our study provides strong evidence supporting the feasibility of inducing hepatocyte differentiation from skin EPCs. These findings underscore the potential of EPCs as a valuable resource in regenerative medicine, paving the way for novel cell-based therapies for liver diseases and their application as in vitro metabolic models for hepatocytes.

## Data Availability

Data sharing is not applicable to this article as no datasets were generated or analyzed during the current study.

## Supporting Information

Supporting Information Table S1. Sequences of oligonucleotide primers used for qPCR.
